# Implication of low dose sacubitril/valsartan

**DOI:** 10.1002/clc.23561

**Published:** 2021-02-04

**Authors:** Teruhiko Imamura

**Affiliations:** ^1^ Second Department of Internal Medicine University of Toyama Toyama Japan

To the editor

The PARADIGM‐HF trial demonstrated that sacubitril/valsartan had an advantage in improving mortality and morbidity over enalapril in patients with systolic heart failure.^1^ However, not many patients can achieve the target dose as recommended in the PARADIGM‐HF trial in the real‐world practice dominantly due to hypotension. Pandey et al. demonstrated that even the lower than the standard dose of sacubitril/valsartan reduced NT‐proBNP maintaining serum potassium and creatinine levels.^2^ Several concerns should improve their findings.

Although statistically not significant, there are numerically several differences in baseline characteristics, including systolic blood pressure, diuretic dose, and beta‐blocker dose, among the three groups stratified by the dose of sacubitril/valsartan.^2^ It would be required to adjust for these differences to investigate the independent impact of very low dose sacubitril/valsartan, although a small sample size would not permit such an adjustment.

Although the patients with very low dose sacubitril/valsartan enjoyed significant improvement in NT‐proBNP, there was no control group,^2^ which might also have achieved similar improvement without sacubitril/valsartan. It would be better to compare their endpoint with a control group without sacubitril/valsartan.

The doses of diuretics could not be reduced and the dose of mineralocorticoid was rather reduced in the patients with very low dose sacubitril/valsartan.^2^ In addition to the decrease in NT‐proBNP level, longer‐term outcomes including cardiac reverse remodeling in such a cohort remain the future concerns.

## CONFLICT OF INTEREST

None.
